# Atherosclerotic plaque, cardiovascular risk, and lipid-lowering strategies: a narrative review

**DOI:** 10.3389/fcvm.2025.1659228

**Published:** 2025-12-16

**Authors:** Frankie Chor-Cheung Tam, Min-Qing Lin, Tsun-Ho Lam, Hung-Fat Tse, Chun-Ka Wong

**Affiliations:** 1 Cardiology Division, Department of Medicine, School of Clinical Medicine, Li Ka Shing Faculty of Medicine, The University of Hong Kong, Hong Kong, Hong Kong SAR, China; 2 Cardiology Division, Department of Medicine, Queen Mary Hospital, Hong Kong, Hong Kong SAR, China; 3 Cardiac and Vascular Center, University of Hong Kong Shenzhen Hospital, Shenzhen, China; 4 Center for Translational Stem Cell Biology, Hong Kong, Hong Kong SAR, China

**Keywords:** atherosclerosis, low density lipoprotein, lipoprotein (a), statin, ezetimibe, proprotein convertase subtilisin/kexin type 9 inhibitors

## Abstract

Atherosclerosis, driven primarily by cumulative exposure to low-density lipoprotein cholesterol (LDL-C), is the major cause of atherosclerotic cardiovascular disease (ASCVD). This narrative review examines the pathogenesis of atherosclerosis, linking risk factors, inflammatory pathways, and lipid abnormalities to the formation and progression of atheromatous plaques. Plaque characteristics such as volume, lipid content, fibrous cap thickness, and minimum lumen area are closely associated with cardiovascular outcomes, particularly the risk of major adverse cardiac events (MACEs). Intensive LDL-C lowering through statins, ezetimibe, PCSK9 inhibitors, and emerging agents like bempedoic acid has demonstrated clear benefits in regressing plaques, stabilizing their morphology, and significantly reducing cardiovascular risks. Despite guideline recommendations advocating intensive lipid-lowering strategies, real-world practice reveals considerable gaps, with many high- and very-high-risk patients failing to achieve LDL-C targets. Contributing factors include poor adherence, underuse of combination therapies, and treatment inertia. Early detection and preemptive management of subclinical atherosclerosis, particularly among younger individuals, are gaining attention as strategies to intercept the progression of disease before clinical events occur. Moreover, elevated lipoprotein(a) levels are increasingly recognized as an independent causal factor for ASCVD, and ongoing trials are evaluating specific Lp(a)-lowering therapies. Overall, optimizing lipid management through intensive, early intervention, patient adherence, and personalized treatment approaches holds the key to reducing the global burden of ASCVD. Addressing residual risks and refining early detection strategies will further advance the prevention and management of this chronic, progressive vascular disease.

## 1 Introduction

Atherosclerosis is a chronic disease originating from deposition of atheromatous plaques inside arterial walls, leading to lumen stenosis and hardening of arteries. Low-density lipoprotein cholesterol (LDL-C) is a causal and cumulative factor in the development of atherosclerosis and subsequent atherosclerotic cardiovascular disease (ASCVD) (1, 2). This narrative review describes the role of atherosclerotic plaque in the onset of ASCVD, relationship between LDL-C level, plaque characteristics, and cardiovascular outcomes as well as clinical implications from the interrelation. However, discrepancy exists between guideline-recommended LDL-C targets and real-world practice for primary and secondary prevention of ASCVD (2). This review also reveals challenges in real-world lipid management and looks forward to optimal atherosclerotic management, suggesting that early detection and treatment of subclinical atherosclerosis at younger age or intensive lipid-lowering strategy is necessary to reduce ASCVD risks.

## 2 Atherosclerosis

### 2.1 Pathogenesis and risk factors

The pathogenic process of atherosclerosis begins with endothelial cell dysfunction, leading to the accumulation and oxidation of LDL-C particles, activation of endothelial cells, and recruitment of monocytes into the intima. In the early stage of atherogenesis, macrophages differentiated from bound monocytes engulf the oxidized LDL and form foam cells. Immune cells such as T cells also contribute to the progression of fatty streak formation. Subsequently, smooth muscle cells from the media migrate and proliferate in the intima, the extracellular matrix such as collagen degrades, and necrosis and calcification develop, resulting in reduced stability of atherosclerotic plaques and eventual rupture (3). Finally, platelets become activated and thrombus formation occurs in the advanced stage of atherosclerosis (3, 4).

**Figure 1:**
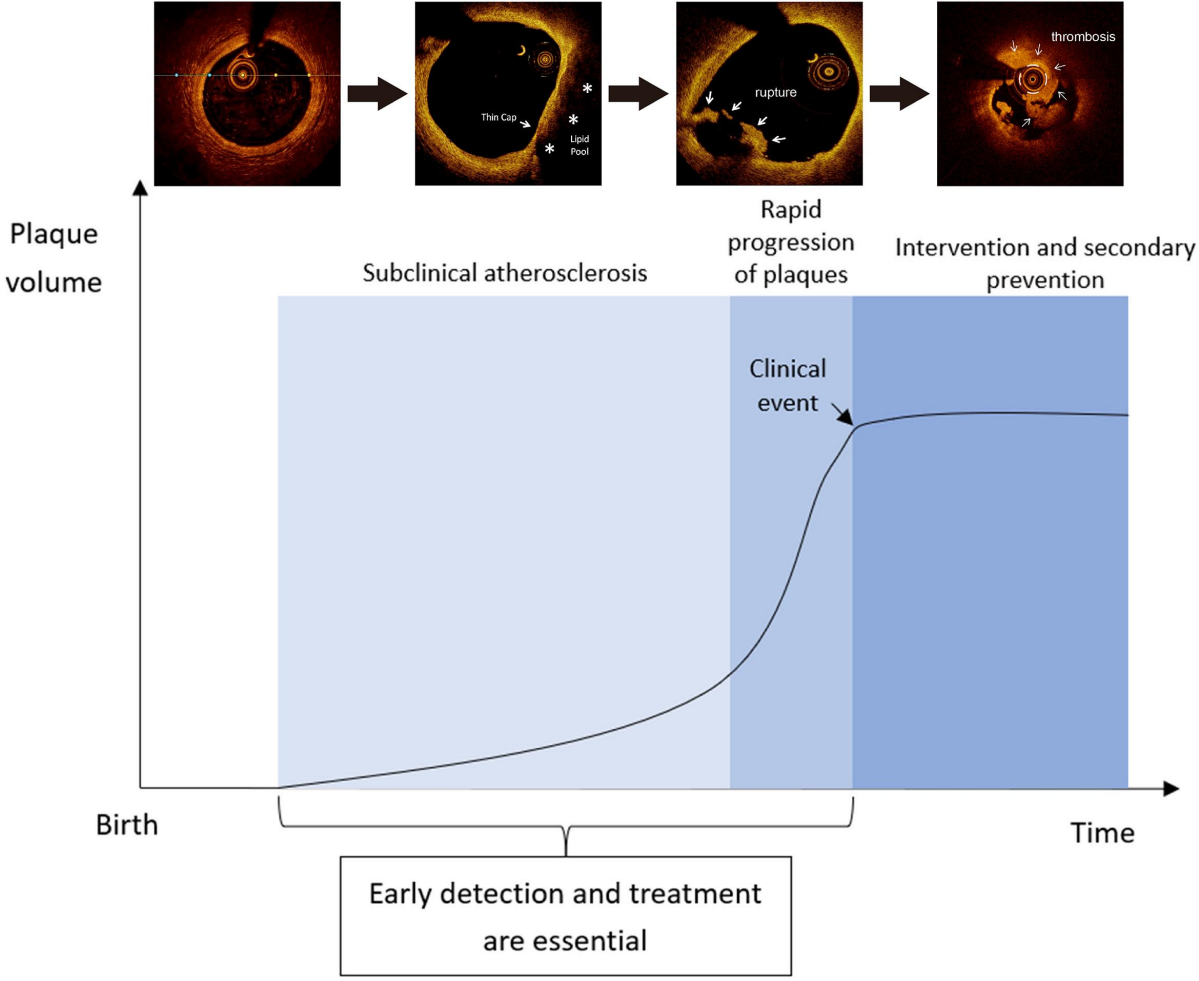
Histological features of atherosclerosis at different stages (11).

Oxidized LDL has conventionally been considered as the primary driver of atherogenesis; however, recent evidence suggests that aggregated LDL associated with proteoglycan, or adaptive immune responses to native LDL, may also be involved in the mechanisms of plaque formation (5). Another factor linked to atherosclerosis is inflammation. Studies have shown that angiotensin II and adaptive T cell immunity, which participate in the pathogenesis of hypertension, can also provide inflammatory pathways for atherosclerosis (6). Biomarkers of inflammation, especially C-reactive protein, are increasingly deemed as predictors of cardiovascular risk (6). It was shown in randomized controlled trial setting that interleukin-1β inhibition in patients with history of myocardial infarction and raised high-sensitive C-reactive protein may potentially reduce subsequent cardiovascular events (7). Clonal hematopoiesis of indeterminate potential, which refers to age-related clonal expansion of blood stem cells with mutations linked to hematologic cancers, is a novel risk factor for inflammation-mediated atherosclerosis and adverse cardiovascular outcomes (6, 8). Microplastics and nanoplastics are also emerging as a potential risk factor for atherosclerosis through activating inflammatory pathways (9). Exposure to the above risk factors may alter the homeostatic properties of the endothelial monolayer, facilitating the initiation of atherogenesis (6). Continued accumulation of lipid and lipid-engorged cells allows the progression of atherosclerotic plaques, many of which will further develop calcification due to dysregulated deposition and impaired clearance of lipids, leading to the potential for rupture and thrombosis (Figure 1) (10, 11).

**Figure 2:**
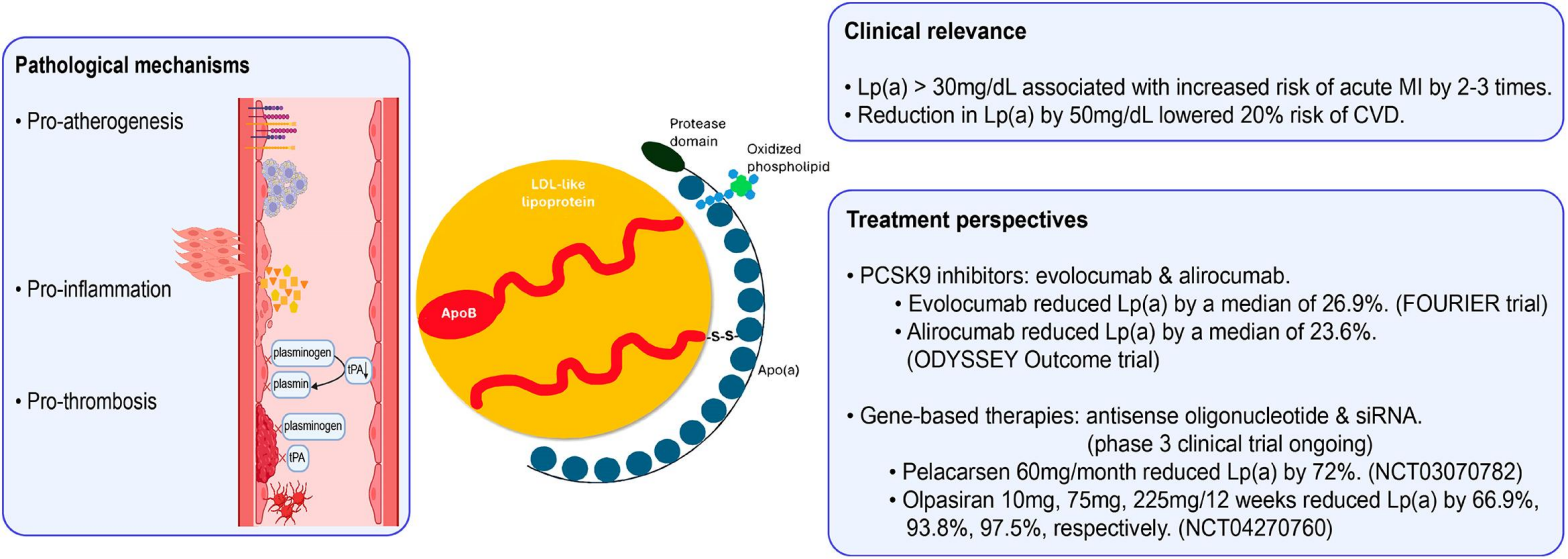
Role of lipoprotein(a) in the atherosclerotic process (12). Apo(a), apolipoprotein(a); ApoB, apolipoprotein B; hs-CRP, high-sensitivity C-reactive protein; IL-6, interleukin 6; LDL, low-density lipoprotein; TNF-α, tumor necrosis factor alpha. Created using Biorender.

Lipoprotein(a) [Lp(a)] has been increasingly recognized to be a contributor to the development of atherosclerosis via several proposed mechanisms (Figure 2) (12). First, Lp(a) may have a pro-atherogenic effect, stimulating the formation of foam cells, proliferation of smooth muscle cells, and production of adherence molecules in endothelial cells of arteries (13). Second, it competes with plasminogen for binding sites on endothelial cells, resulting in antifibrinolytic and pro-thrombotic effects (14). Third, Lp(a) may stimulate inflammatory cytokines, which include interleukin-6 and tumor necrosis factor-α, possibly increasing the risk of inflammation and thus atherosclerosis (15). Recent research suggests that, although the underlying pathophysiology is not fully understood, an increase in Lp(a) level is an independent risk factor for coronary heart disease (CHD) (12).

### 2.2 Plaque characteristics and cardiovascular events

Once established, atherosclerotic plaques continue to encroach upon the arterial lumen, leading to the formation of flow-limiting lesions and ischemia, and subsequently plaque rupture (10), which is the most common cause of acute thrombosis of coronary arteries leading to myocardial infarction (MI) (16, 17). Recent studies using serial angiographic data demonstrated that progression of atherosclerotic plaques can be rapid, possibly due to plaque disruption and subsequent thrombotic organization shortly (i.e., within 1–3 months) before the onset of an acute clinical event, such as MI (18, 19). Thus, early detection and treatment of subclinical atherosclerosis at a younger age have been increasingly emphasized to modify plaque progression, reduce rupture, and prevent atherosclerotic clinical events (18).

A number of studies using coronary angiography (Table 1) revealed that plaque progression in terms of luminal stenosis shortly before or at the onset of an acute MI, recurrent MI, or major adverse cardiac event (MACE, which is generally defined as the composite of cardiac death, non-fatal MI, unstable angina, or coronary revascularization) (20–24). One study in Japan demonstrated the process of plaque progression in coronary artery disease using four serial coronary arteriograms within 1 year (25). Among 36 patients, 14 (39%) had vessels with marked plaque progression and a sudden surge in the mean stenosis observed from the third (46 ± 13%) to the fourth arteriogram (88 ± 10%); 71% of these patients sustained acute coronary syndrome (25). In contrast, only three (14%) of the 22 patients with gradual progression of stenosis throughout four arteriograms had an acute coronary event (25). Taken together, current coronary angiography studies suggest that rapid, substantial plaque progression is a critical predictor of plaque rupture and subsequent MI or other MACEs.

**Table 1:** Coronary angiography studies on changes in luminal stenosis from atherosclerosis to an acute event.

Study	No. of patients/lesions	Time of measurement	Mean (SD) diameter stenosis (%)
Ojio et al. (20)	20	6–18 months before MI	30 (18)
	20	≤1 week before MI	71 (12)
Glaser et al. (21)	157	At the time of initial PCI	41.8 (20.8)
		At the onset of a recurrent event (follow-up, 1 year)	83.9 (13.9)
PROSPECT (22)	74	Baseline	32.3 (20.6)
		At the onset of a MACE (median follow-up, 3.4 years)	65.4 (16.3)
Zaman et al. (23)	34	>3 months before MI	36.5 (20.6)
	7	≤3 months before MI	59.1 (31.5)
PROSPECT II (24)	66	Baseline	46.9 (15.9)
		At the onset of a MACE (median follow-up, 3.7 years)	68.4 (17.7)

MACE, major adverse cardiac event; MI, myocardial infarction; PCI, percutaneous coronary intervention; SD, standard deviation.

In addition to stenosis, features of plaque morphology, such as plaque burden, minimum lumen area (MLA), fibrous cap thickness (FCT), lipid burden, and lipid arc, have also been shown to be associated with the risk of MACEs in multiple studies using optical coherence tomography (OCT), intravascular ultrasound (IVUS), or near-infrared spectroscopy (Table 2) (22, 24, 26–28). In the PROSPECT study (22), a plaque burden ≥70% (hazard ratio [HR], 5.03; 95% confidence interval [CI], 2.51–10.11; *P* < 0.001), an MLA ≤4.0 mm^2^ (HR, 3.21; 95% CI, 1.61–6.42; *P* = 0.001), and a classification of thin-cap fibroatheromas (TCFAs; HR, 3.35; 95% CI, 1.77–6.36; *P* < 0.001) were significant risk factors for recurrent MACEs related to non-culprit lesions. The LRP study (26) showed that each 100-unit increase in the maximum 4-mm Lipid Core Burden Index (maxLCBI_4 mm_) significantly elevated the risk of non-culprit MACEs, with unadjusted and adjusted HRs on a patient level 1.21 (95% CI, 1.09–1.35; *P* = 0.0004) and 1.18 (95% CI, 1.05–1.32; *P* = 0.0043), respectively; and unadjusted HR on a lesion level 1.45 (95% CI, 1.30–1.60; *P* < 0.0001) (26). Likewise, the PROSPECT II study (24) showed that a high lipid burden (maxLCBI_4 mm_ ≥ the upper quartile of all non-culprit lesions) and a large plaque burden (≥70%) were independent predictors of non-culprit MACEs (24). In the CLIMA study (27) of 1,776 non-culprit plaques, an MLA <3.5 mm^2^ (HR, 2.1; 95% CI, 1.1–4.0), FCT <75 mm (HR, 4.7; 95% CI, 2.4–9.0), lipid arc circumferential extension >180° (HR, 2.4; 95% CI, 1.2–4.8), and the presence of OCT-defined macrophages (HR, 2.7; 95% CI, 1.2–6.1) were associated with an elevated risk of cardiac death or target segment MI (27). The recent COMBINE OCT-FFR study (28) provided further insights into the classification of lipid-rich plaque (LRP) lesions that increased the risk of MACEs. LRP lesions were associated with a higher risk of MACEs (HR, 3.9; 95% CI, 0.9–16.5; log-rank *P* = 0.049) than non-LRP lesions; however, TCFAs, which accounted for one-third of LRP lesions, had a significantly higher risk of MACEs compared with thick-cap fibroatheromas (ThCFAs; HR, 3.8; 95% CI, 1.5–9.5; *P* < 0.01) as well as non-LRP lesions (HR, 7.7; 95% CI, 1.7–33.9; *P* < 0.01) (25). ThCFAs and non-LRP lesions were not significantly different in terms of the risk of MACEs (HR, 2.0; 95% CI, 0.42–9.7; *P* = 0.38) (28).

**Table 2:** Characteristics of selected studies that investigated relationships between plaque morphology and the risk of cardiac events.

Study	No. of patients	Follow-up (months)	Primary endpoint	Imaging tool	Key findings
PROSPECT (22)	697	40.8	Culprit and non-culprit MACEs	IVUS	Plaque burden ≥70% (vs. <70%), MLA ≤4.0 (vs. >4.0 mm^2^), and TCFA (vs. ThCFA) were significant risk factors for recurrent MACEs related to non-culprit lesions
LRP (26)	1271	24	Non-culprit MACEs	NIRS-IVUS	Each 100-unit increase in maxLCBI_4mm_ significantly elevated the risk of non-culprit MACEs
CLIMA (27)	1,003	12	Cardiac death and target segment MI	OCT	MLA <3.5 (vs. ≥3.5 mm^2^), FCT <75 (vs. ≥75 µm), lipid arc circumferential extension >180° (vs. ≤180°), and the presence of OCT-defined macrophages were associated with an elevated risk of cardiac death or target segment MI
PROSPECT II (24)	898	44.4	Non-culprit MACEs	NIRS-IVUS	MaxLCBI_4 mm_ ≥ 324.7 (vs. <324.7) and plaque burden ≥70% (vs. <70%) were independent predictors of non-culprit MACEs
COMBINE OCT-FFR (28)	390	18	Non-culprit MACEs	OCT + FFR	LRP (vs. non-LRP) lesions were associated with a higher risk of MACEs; LRP-TCFA (FCT ≤65 µm; lipid arc >90°) had a significantly higher risk of MACEs vs. LRP-ThCFA (FCT >65 µm) and non-LRP lesions; ThCFAs and non-LRP lesions were not significantly different in terms of the risk of MACEs

FCT, fibrous cap thickness; FFR, fractional flow reserve; IVUS, intravascular ultrasound; LRP, lipid-rich plaque; MACE, major adverse cardiac event; maxLCBI_4 mm_, maximum 4 mm Lipid Core Burden Index; MI, myocardial infarction; MLA, minimum lumen area; NIRS, near-infrared spectroscopy; OCT, optical coherence tomography; TCFA, thin-cap fibroatheroma; ThCFA, thick-cap fibroatheroma.

## 3 Management of atherosclerosis

Lipid-lowering therapy targeting reductions in LDL-C levels remains the primary medicinal intervention for atherosclerotic cardiovascular disease (ASCVD). Statins are the most widely used lipid-lowering agents. Ezetimibe (29) and proprotein convertase subtilisin/kexin type 9 (PCSK9) inhibitors (30, 31) are feasible treatment options to further reduce LDL-C levels. Bempedoic acid is a recently developed lipid-lowering agent that can be used in statin-intolerant patients (32). The sections below discuss the clinical data on relationships between reductions in LDL-C levels with lipid-lowering therapies, the morphology of coronary atherosclerotic plaques, and the risk of cardiovascular disease.

### 3.1 Correlation between achieved LDL-C levels and plaque characteristics

**Figure 3:**
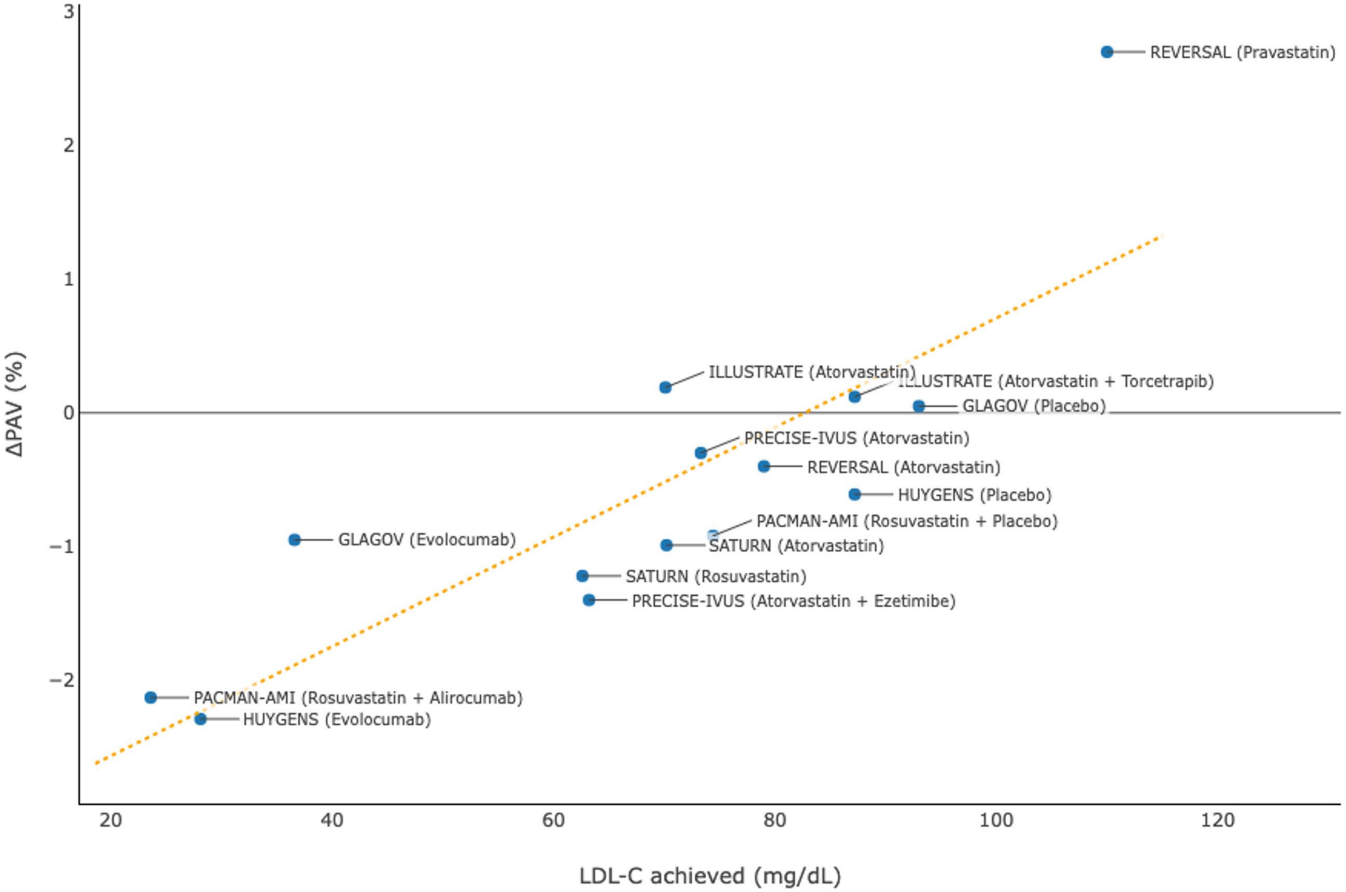
Association between achieved levels of low-density lipoprotein cholesterol (LDL-C) and change in percent atheroma volume (PAV) in clinical trials using intravascular ultrasonography.

Multiple clinical studies (29–31, 33–37) have been conducted to investigate the effects of intensive lipid-lowering therapies, including high-dose statins and PCSK9 inhibitors, on reducing the progression of atherosclerotic plaques among patients with coronary artery disease (at least one vessel with stenosis ≥20%; target segment with stenosis ≤50%) using IVUS imaging (Table 3). Clinical trial results were identified from PubMed and pooled data from these studies demonstrated that a lower level of achieved LDL-C was associated with more substantial regression of atherosclerotic plaques in terms of percent atheroma volume (Figure 3).

**Table 3:** Prospective and randomized trials on the effects of intensive lipid-lowering therapies on the regression of coronary atherosclerotic plaques using intravascular ultrasonography.

Study	No. of patients	Follow-up (months)	Study arm			Comparator arm		
			Treatment	LDL-C (mg/dl)	Change in PAV (%)	Treatment	LDL-C (mg/dl)	Change in PAV (%)
REVERSAL (33)	502	18	Atorvastatin	79	+0.6	Pravastatin	110	+1.9
ILLUSTRATE (35)	910	24	Atorvastatin + Torcetrapib	70.1	+0.12	Atorvastatin	87.2	+0.19
SATURN (36)	1,039	24	Rosuvastatin	62.6	−1.22	Atorvastatin	70.2	−0.99
PRECISE-IVUS (29)	202	9–12	Atorvastatin + Ezetimibe	63.2	–1.4	Atorvastatin	73.3	–0.3
GLAGOV (37)	968	19	Evolocumab	36.6	–0.95	Placebo	93.0	+0.05
HUYGENS (30)	161	12	Evolocumab	28.1	–2.29	Placebo	87.2	–0.61
PACMAN-AMI (31)	300	12	Rosuvastatin + Alirocumab	23.6	–2.13	Rosuvastatin + Placebo	74.4	–0.92

LDL-C, low-density lipoprotein cholesterol; PAV, percent atheroma volume.

**Figure 4:**
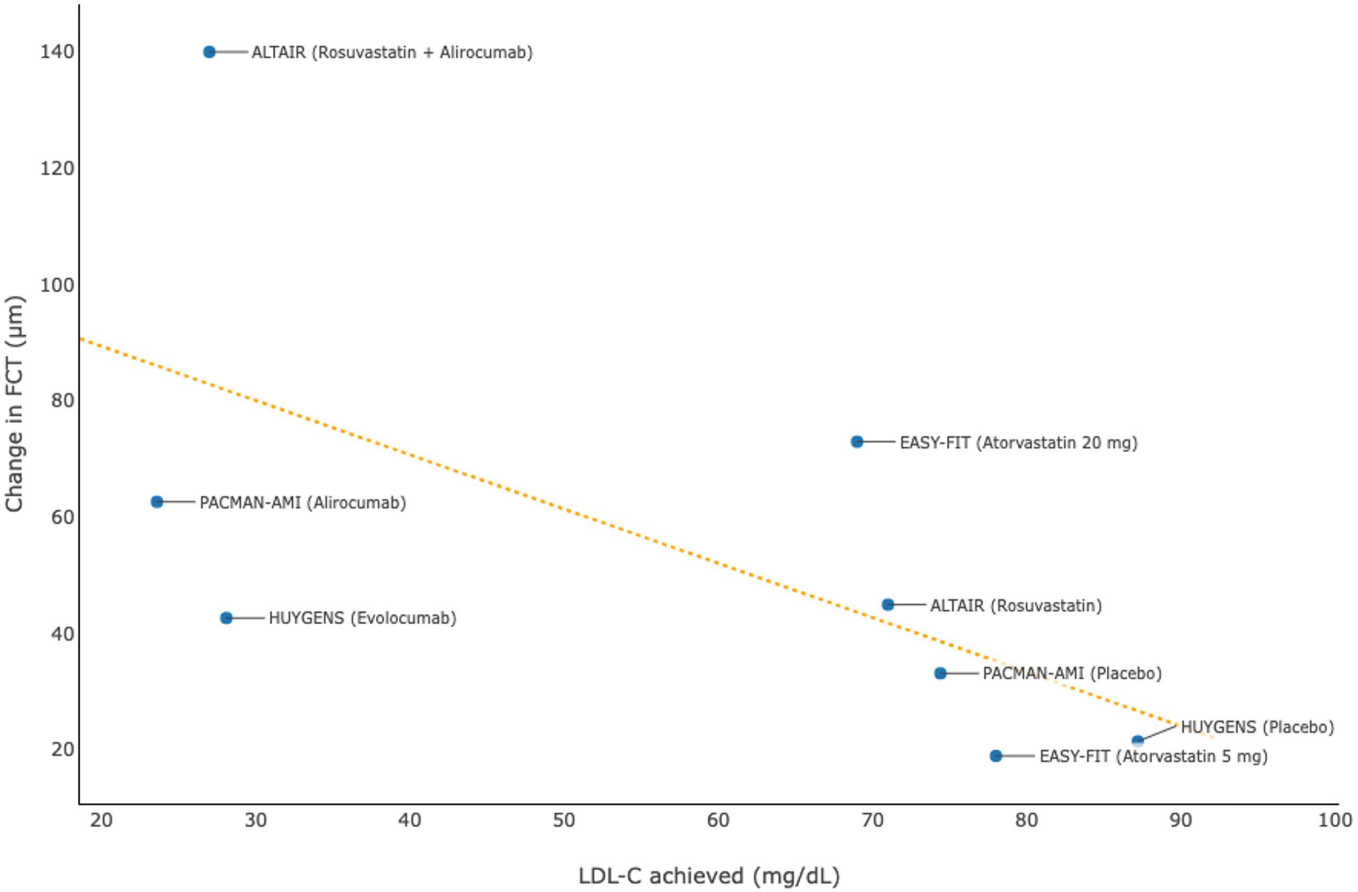
Association between achieved levels of low-density lipoprotein cholesterol (LDL-C) and change in fibrous cap thickness (FCT) in clinical trials using optical coherence tomography.

Studies using OCT (30, 31, 38–40) also showed that intensive lipid-lowering therapies were associated with favorable changes in plaque composition and microstructure, which included evaluation of FCT, lipid burden, MLA, and macrophage accumulation among patients with acute coronary syndrome (Table 4). The aggregated evidence, particularly from the HUYGENS (30) and PACMAN-AMI (31) studies on PCSK9 inhibitors, revealed that a lower level of LDL-C achieved was associated with a more marked increase in FCT (Figure 4), suggesting a higher degree of stabilization of atherosclerotic plaques.

**Table 4:** Prospective and randomized trials on the effects of intensive lipid-lowering therapies on the stabilization of coronary plaques using optical coherence tomography.

Study	No. of patients	Follow-up (months)	Study arm						Comparator arm					
			Treatment	LDL-C achieved (mg/dl)^a^	Change in FCT (µm)	Change in lipid burden (degree)	Change in MLA (mm^2^)	Macrophage accumulation^b^	Treatment	LDL-C achieved (mg/dl)^a^	Change in FCT (µm)	Change in lipid burden (degree)	Change in MLA (mm^2^)	Macrophage accumulation^b^
EASY-FIT (38)	60	12	Atorvastatin 20 mg qd	69	+73	−50	−0.05	−4.5	Atorvastatin 5 mg qd	78	+19	−10	−0.09	−2.0
ALTAIR (40)	24	9	Rosuvastatin + Alirocumab	27	+140	−26.2% (lipid index)	NR	−28.4	Rosuvastatin	71	+45	−2.8% (lipid index)	NR	−10.2
HUYGENS (30)	161	12	Evolocumab	28.1	+42.7	−57.5	NR	−3.17	Placebo	87.2	+21.5	−31.4	NR	−1.45
PACMAN-AMI (31)	300	12	Alirocumab	23.6	+62.67	−79.42 (LCBI)	NR	−25.98	Placebo	74.4	+33.19	−37.60 (LCBI)	NR	−15.95

FCT, fibrous cap thickness; LCBI, Lipid Core Burden Index (maximum 4-mm); MLA, minimum lumen area; NR, not reported; qd, once daily.

a Median levels of low-density lipoprotein cholesterol (LDL-C) were reported in the EASY-FIT and ALTAIR studies; mean levels of LDL-C were reported in the HUYGENS and PACMAN-AMI studies.

b Accumulation grades were reported in EASY-FIT and ALTAIR; data on angular extension were reported in PACMAN-AMI; macrophages indexes were reported in HUYGENS.

Taken together, clinical trials using IVUS and OCT have consistently shown that intensive reductions in LDL-C levels are associated with regression and stabilization of atherosclerotic plaques in patients with CHD.

Although we must acknowledge potential limitations of using imaging as a surrogate endpoint, such as imperfect correlation with clinical outcomes and measurement variability, it remains a valuable tool for assessing structural changes and providing insight into plaque progression in the process of atherosclerosis.

### 3.2 Correlation between achieved LDL-C levels and cardiovascular outcomes

**Figure 5:**
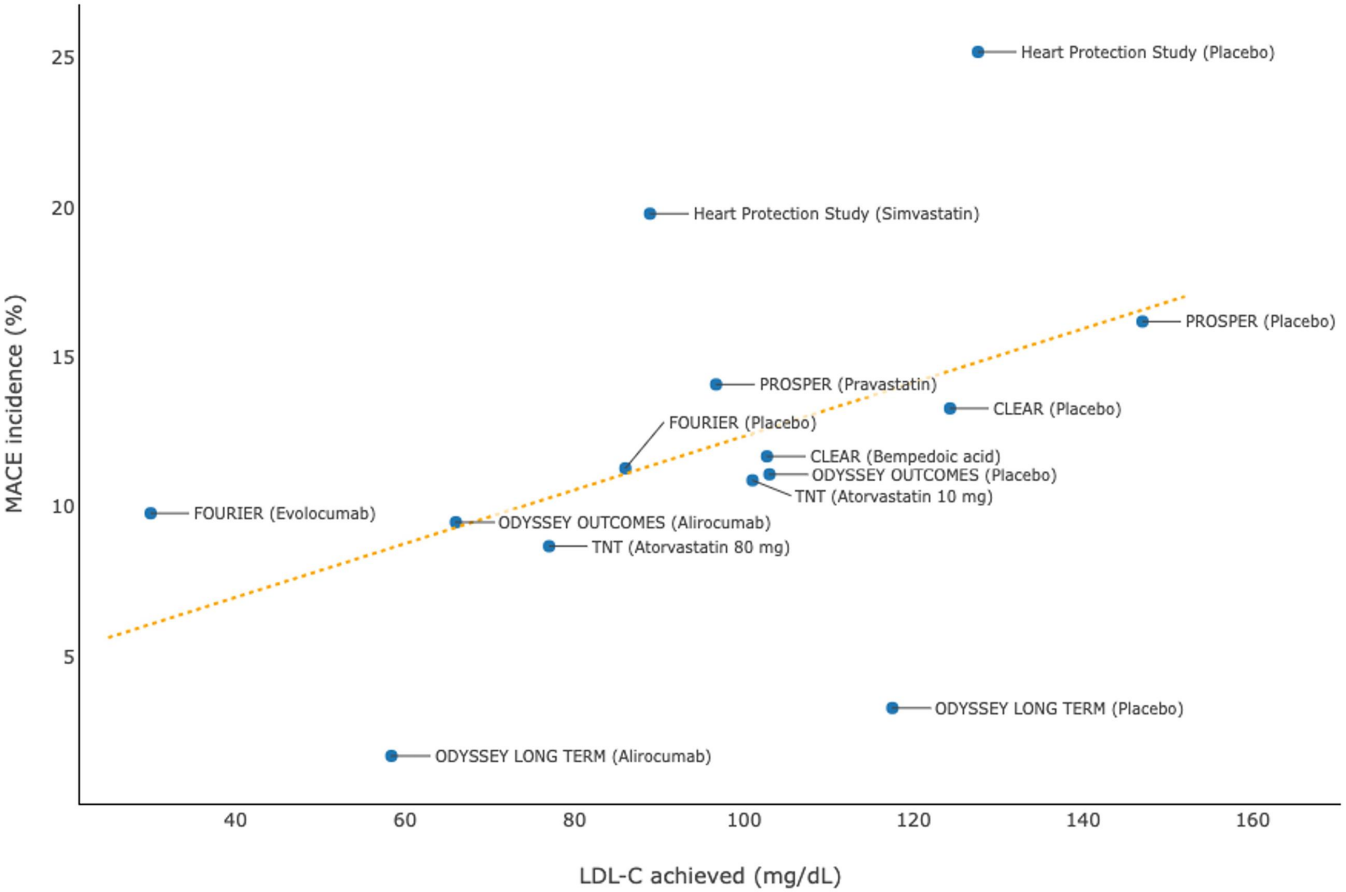
Association between achieved levels of low-density lipoprotein cholesterol (LDL-C) and incidences of major adverse cardiovascular events (MACEs): pooled data from randomized controlled trials.

A systematic review and meta-regression analysis of 17 prospective studies of dyslipidemia therapies showed that a 1% decrease in the mean percent atheroma volume resulted from these treatments was associated with a 20% reduction in the risk of MACEs (adjusted odds ratio, 0.82; 95% CI, 0.70–0.95; *P* = 0.011) (41). In line with the beneficial effects on modifying the morphology of atherosclerotic plaques, intensive lipid-lowering therapies significantly reduce the risk of cardiovascular disease among patients with or without a history of CHD. A number of large-scale, long-term randomized controlled trials (Table 5) demonstrated that intensive reductions in levels of LDL-C using high-dose statins, PCSK9 inhibitors, or bempedoic acid significantly reduced the risk of first-onset or recurrent MACEs, which were generally referred to as the composite of cardiovascular death, non-fatal MI, non-fatal stroke, unstable angina, or coronary revascularization (32, 42–50). Pooled data from each randomized treatment arm showed that a lower level of LDL-C achieved was strongly associated with a lower incidence of MACEs (Figure 5). Likewise, greater absolute reductions in LDL-C levels resulted from intensive lipid-lowering therapies compared with controls were associated with more substantial reductions in the relative risk of MACEs (i.e., lower HRs were reported in the randomized controlled trials), suggesting that there is a dose-dependent effect of LDL-C reduction on lowering the cardiovascular risk, regardless of the history of CHD (32, 42–50).

**Table 5:** Randomized controlled trials on the efficacy of intensive lipid-lowering therapies in reducing the cardiovascular risk in high-risk patients (primary prevention) or patients with a history of coronary heart disease (secondary prevention).

Study	Level of prevention	No. of patients	Follow-up (years)^a^	Study arm			Comparator arm			Absolute reduction in LDL-C (mg/dl)	Relative risk reduction in MACE (%)
				Treatment	LDL-C achieved (mg/dl)^b^	MACE incidence (%)^c^	Treatment	LDL-C achieved (mg/dl)^b^	MACE incidence (%)^c^		
PROSPER (42)	Secondary	5,804	3.2	Pravastatin	96.7	14.1	Placebo	146.5	16.2	49.8	15
Heart Protection Study (43)	Secondary	20,536	4.8–5.0	Simvastatin	88.9	19.8	Placebo	127.6	25.2	38.7	24
TNT (44)	Secondary	10,001	4.9	Atorvastatin 80 mg qd	77.0	8.7	Atorvastatin 10 mg qd	101.0	10.9	24.0	22
JUPITER (45)	Primary	17,802	1.9	Rosuvastatin	55.0	1.6	Placebo	109.0	2.8	54.0	44
ODYSSEY LONG TERM (46)	Secondary	2,341	1.5	Alirocumab	58.4	1.7	Placebo	117.5	3.3	59.1	48
HOPE-3 (47)	Primary	12,705	5.6	Rosuvastatin	95.0	4.4	Placebo	124.5	5.7	29.5	25
OSLER (48)	Primary	4,465	∼1.0	Evolocumab + SOC	48.0	1.0	SOC	Not reported	2.2	73.0^d^	53
FOURIER (49)	Secondary	27,564	2.2	Evolocumab	30.0	9.8	Placebo	Not reported	11.3	56.0^d^	15
ODYSSEY OUTCOMES (50)	Secondary	18,924	2.8	Alirocumab	66.0	9.5	Placebo	103.0	11.1	37.0	15
CLEAR (32)	Secondary	13,970	3.4	Bempedoic acid	102.7	11.7	Placebo	124.3	13.3	21.6	13

qd, once daily; SOC, standard of care.

a Mean durations of follow-up were reported in the PROSPER, Heart Protection, and ODYSSEY LONG TERM studies; the remaining studies reported median durations of follow-up.

b Median levels of low-density lipoprotein cholesterol (LDL-C) were reported in the JUPITER, OSLER, and FOURIER studies; the remaining studies reported mean levels of LDL-C.

c A major adverse cardiovascular event (MACE) was generally defined as the composite of cardiovascular death, non-fatal myocardial infarction, non-fatal stroke, unstable angina, or coronary revascularization.

d In OSLER and FOURIER, reductions in LDL-C were means, whereas achieved LDL-C levels were medians.

### 3.3 Guideline recommendations for intensive lipid-lowering treatment

In a consensus statement from the European Atherosclerosis Society (EAS) (4), LDL-C is recognized as both a causal and a cumulative factor for the initiation and progression of ASCVD. The lower the LDL-C level achieved with lipid-lowering agents, the greater the clinical benefit amassed (4). Both relative and absolute risk reduction in major cardiovascular events resulting from lowering LDL-C will depend on a patient's baseline LDL-C level, the absolute magnitude of LDL-C reduction, and the duration of lipid-lowering treatment (4).

More recently, the guidelines for the management of dyslipidemia established jointly by the European Society of Cardiology (ESC) and the EAS have articulated the dose-dependent effect of LDL-C-lowering agents on reducing the risk of ASCVD (51). The guidelines have also highlighted that the primary goal of targeted lipid management is to reduce atherosclerotic risk by markedly lowering LDL-C to levels that were attained in randomized controlled trials of PCSK9 inhibitors. In patients at high or very high cardiovascular risk, reducing LDL-C to as low a level as possible or a minimum 50% reduction from the baseline LDL-C level, along with achieving the tailored goal is suggested (51).

To achieve the ESC/EAS-recommended goals for LDL-C in patients at very high cardiovascular risk, even treatment with high-dose statins is often insufficient (52). Ray et al. proposed that the treatment paradigm for these patients should be shifted from an “intensive statin therapy first” approach to an “intensive lipid-lowering combination therapy” approach using ezetimibe or a PCSK9 inhibitor as an adjunct agent to facilitate effective LDL-C lowering and thus cardiovascular risk reduction (52).

## 4 Barriers to real-world lipid management

### 4.1 Discrepancy between guideline recommendations and routine clinical practice

In view of the evidence-based cardioprotective benefits, intensive LDL-C–lowering therapy has been recommended for patients at risk of ASCVD (4, 51). However, achieving and maintaining guideline-recommended LDL-C goals in real-world practice remains a therapeutic challenge. A retrospective cohort study in the U.S. revealed that nearly 50% of patients aged ≥65 years discontinued statin treatment within the first year of the initial prescription, with a substantial decline in the adherence rate over time (53). Real-world clinical data from Germany (54) and Poland (55) showed that only 20% of very high-risk patients attained the LDL-C goal of <1.8 mmol/L as recommended by the ESC/EAS at the time of the studies. More recently, the cross-sectional DA VINCI study conducted in 18 European countries found that only 25% and 11% of individuals at high risk and very high risk, respectively, achieved the LDL-C targets for primary prevention, and that only 18% of patients with established ASCVD attained the LDL-C goals (56).

### 4.2 Possible reasons for not achieving the target LDL-C level

There are several possible reasons behind the failure to achieve guideline-recommended LDL-C goals. Non-adherence to lipid-lowering treatment is one major obstacle. Muscle symptoms, such as myalgia, are the most frequent adverse event (AE) that interrupts statin therapy (10). Other factors, such as patient education, complexity of treatment regimens, availability of drugs, reimbursement policies, and physician practice, also affect patient adherence (58). Another obstacle is the low use of guideline-recommended combination therapy for high- and very high-risk patients. The DA VINCI study (56) showed that moderate-intensity statin monotherapy was the most commonly used regimen for primary prevention of ASCVD in patients at high risk (64%) and very high risk (69%). Across all risk categories, merely 9% of patients received ezetimibe in combination with a statin, and 1% of patients received a PCSK9 inhibitor with a statin and/or ezetimibe (56).

Some clinicians and patients have concern about the safety of achieving “extremely low” LDL-C (59). *Post-hoc* analysis of randomized controlled trials involving PCSK9 inhibitors revealed no increased risk of new or recurrent cancer, cataract-related adverse events, hemorrhagic stroke, new-onset diabetes, neurocognitive adverse events, muscle-related events, or non-cardiovascular death, were found in patients with LDL-C <0.5 mmol/L (60).

### 4.3 Optimizing the management of ASCVD

The benefits of intensive LDL-C–lowering observed in clinical trials will only materialize in real-world practice if patients adhere to treatment. Patients who are suspected to have statin intolerance should consider careful statin re-challenge, because continued statin use after experiencing an AE remains effective in reducing the risk of cardiovascular events and all-cause mortality, especially in high-risk patients (61–63). Lipid-lowering combination therapy should be promoted to facilitate the attainment of guideline-recommended LDL-C goals in patients at high and very high risk (52). Treatment adherence should be enhanced by improving health literacy. Even after achieving and maintaining LDL-C goals, patients should address the long-term residual risk of cardiovascular events by adhering to optimal management of comorbidities and recommended lifestyle modifications.

### 4.4 Emerging evidence on functionality of high-density lipoprotein (HDL)

While intensive LDL-C lowering remains a cornerstone of atherosclerotic cardiovascular disease prevention, emerging evidence indicates that considerable residual risk persists even after achieving very low LDL-C levels. This observation suggests that lipid-related risk extends beyond LDL-C concentration alone. Increasing recognition of dysfunctional HDL challenges the traditional paradigm that higher HDL-C invariably confers protection, underscoring that lipoprotein functionality may be as important as its circulating levels (57). Oxidative and inflammatory modifications can render HDL pro-atherogenic, impairing its cholesterol efflux, antioxidant, and anti-inflammatory properties (57). These findings collectively question whether further LDL-C reduction alone can meaningfully address residual risk. A more holistic approach that targets the restoration of lipoprotein quality and function may offer greater benefit in mitigating cardiovascular disease burden.

## 5 Future development

### 5.1 Preemptive management of subclinical atherosclerosis

With a slow progression, atherosclerosis begins decades before the advent of clinical symptoms, which are often related to luminal stenosis or thrombotic obstruction. Studies have shown that subclinical atherosclerosis occurs and progresses early in life (10). In the PESA prospective cohort study of asymptomatic participants (mean age, 45.8 years) from a Spanish bank (64), subclinical atherosclerosis was detected using ultrasound and computed tomography (CT) in 63% of the overall cohort (71% for males; 48% for females). An analysis (65) of Framingham Heart Study revealed that participants with no established risk factors at 50 years of age had a minimal lifetime risk for ASCVD (51.7% for men; 39.2% for women) and a long survival (median, 30 years for men; 36 years for women). These data suggest that addressing subclinical atherosclerosis and other risk factors at younger ages before symptom onset should be an emerging paradigm for the prevention of ASCVD.

An early, intensive reduction in LDL-C levels during young adulthood is increasingly considered an effective approach for the prevention of ASCVD via minimization of the progression of atherosclerotic plaque (66, 67). Six-year follow-up data from the PESA cohort study (66) demonstrated that one-third of middle-aged (40–55 years at baseline), asymptomatic individuals had progression of subclinical atherosclerosis, and that the effect of elevated LDL-C on the risk of atherosclerotic progression was more significant in younger participants (age groups, 40–43, 44–47, and ≥48 years; *P* for interaction = 0.04). These findings highlight the importance of strictly controlling risk factors at young ages for the prevention of atherosclerotic progression (66). Recently, a phase IV open-label single-arm ARCHITECT trial utilized a non-invasive quantitative CT scan to assess changes in coronary plaque burden and its morphology in patients who received alirocumab for familial hypercholesterolemia and had no clinical ASCVD (68). After 78 weeks of treatment, the patients demonstrated significant plaque regression (burden reduced from 34.6% to 30.4%; *P* < 0.001) and plaque stabilization (fibro-fatty and necrotic plaque reduced by 3.9% [*P* < 0.001] and 0.6% [*P* < 0.001], respectively) on CT angiography (68). The ongoing PRECAD randomized trial (67) investigates the effect of maintaining LDL-C levels <70 mg/dl, along with rigorous control of blood pressure and glucose, on the risk of new-onset atherosclerosis and/or its progression in individuals aged 20–39 years without known cardiovascular disease. The results will inform the future prospect of primary prevention strategies for ASCVD in young adults.

### 5.2 Lp(a)

Elevated Lp(a) levels have been increasingly recognized as a causal and continuous risk factor for cardiovascular events. It is estimated that each 50 nmol/L increase in the Lp(a) level compared to the median is associated with an approximately 20% surge in the risk of MACEs (69). Despite strong genetic and epidemiological evidence linking elevated Lp(a) to ASCVD risk, no specific Lp(a)-lowering therapy has yet received regulatory approval. While we eagerly anticipate the results of large-scale randomized controlled trials investigating the impact of specific Lp(a)-lowering therapies on the risk of MACEs in patients with ASCVD, including Lp(a)HORIZON on pelacarsen (NCT04023552) and OCEAN(a) on olpasiran (NCT05581303), an extra reduction in LDL-C could be considered to mitigate the residual risk associated with an elevation in Lp(a) at different ages (69). Additionally, reductions in Lp(a) induced by PCSK9 inhibitors may further reduce the risk of cardiovascular events. In a pre-specified analysis of the phase III randomized FOURIER trial, evolocumab reduced the risk of CHD death, MI or urgent revascularization by 23% (HR, 0.77; 95% CI, 0.67–0.88) in patients with a higher-than-median level of Lp(a) at baseline, and by 7% (HR, 0.93; 95% CI, 0.80–1.08; *P*_interaction_ = 0.07) in patients with a Lp(a) level not exceeding the median at baseline (70). In a pre-specified analysis of the phase III randomized ODYSSEY Outcomes trial, a 1 mg/dl reduction in Lp(a) with alirocumab was associated with an HR of 0.994 (95% CI, 0.990–0.999; *P* = 0.0081) for the risk of MACEs (71).

## 6 Conclusion

The formation, evolution, and rupture of atherosclerotic plaque are the major risk factors for the development of ASCVD, which is a critical public health threat worldwide. Plaque progression in terms of luminal stenosis and morphological features is associated with an elevated risk of acute coronary syndrome. Reductions in LDL-C levels have dose-dependent effects on the regression and stabilization of atherosclerotic plaques, as well as the reduction in the risk of cardiovascular disease. The relationships between LDL-C levels, plaque morphology, and clinical outcomes have supported the central role of intensive lipid-lowering therapy in the prevention of ASCVD. Current guidelines recommend that individuals at very high risk should consider combination therapy to facilitate the attainment of the LDL-C goal. In real-world practice, patient adherence is the prerequisite to acquire the clinical benefits of intensive lipid-lowering therapy. Considering the rapidity of plaque progression, subclinical atherosclerosis should be detected, preferably with non-invasive imaging tools, at early stages, facilitating early management of LDL-C levels and prevention of ASCVD in the long term.
